# Differential Cellular Expression of Galectin-1 and Galectin-3 After Intracerebral Hemorrhage

**DOI:** 10.3389/fncel.2019.00157

**Published:** 2019-05-14

**Authors:** Frederick Bonsack, Sangeetha Sukumari-Ramesh

**Affiliations:** Department of Pharmacology & Toxicology, Medical College of Georgia, Augusta University, Augusta, GA, United States

**Keywords:** ICH, Galectin-1, Galectin-3, astrocytes, microglia

## Abstract

Intracerebral hemorrhage (ICH) is a devastating sub-type of stroke with no proven treatment. Given the emerging role of Galectin-1 and Galectin-3 in neuroimmune responses, the objective of the current manuscript is to elucidate hemorrhagic-injury induced modulation and cellular expression of Galectin-1 and Galectin-3 in the brain in a pre-clinical model of ICH. To address this, ICH was induced in male CD1 mice by collagenase injection method. Western blotting as well as Immunofluorescence staining was performed to characterize the temporal expression pattern as well as cellular localization of Galectin-1 and Galectin-3 after ICH. Further, genetic studies were conducted to assess the functional role of Galectin-1 and Galectin-3 in inflammatory response employing a murine macrophage cell line, RAW 264.7. Galectin-1 and Galectin-3 exhibited very profound and increased expression from day 3 to day 7-post-injury, in the perihematomal brain region after ICH in comparison to Sham. Further, Galectin-1 expression was mostly observed in GFAP-positive astrocytes whereas Galectin-3 expression was observed mostly in Iba1-positive microglia/macrophages as well as CD16/32 (M1 microglial/macrophage marker)-positive cells. Moreover, genetic studies revealed a negative regulatory role of both Galectin-1 and Galectin-3 in the release of a proinflammatory cytokine, IL-6 from RAW 264.7 cells depending on the stimulus. Altogether, the present manuscript demonstrates for the first time, increased expression as well as cellular localization of Galectin-1 and Galectin-3 in the perihematomal brain regions after ICH. In addition, the manuscript raises the potential of Galectin-1 and Galectin-3 in modulating glial responses and thereby brain injury after ICH, warranting further investigation.

## Introduction

Intracerebral hemorrhage is a fatal stroke subtype (Qureshi et al., 2001a) that accounts for an in-hospital mortality rate and a disability rate of 40 and 80%, respectively (van Asch et al., 2010). ICH is responsible for 10–15% of all strokes, and the worldwide incidence of ICH is 2 million cases per year (van Asch et al., 2010) with approximately 120,000 cases per year in the United States (Ribo and Grotta, 2006; Broderick et al., 2007; Aguilar and Freeman, 2010). However, the incidence is expected to have doubled by 2050 (Qureshi et al., 2001b) due to aging and the spreading use of anticoagulants (Wang, 2010). Notably, there is no effective treatment for ICH, and the pathophysiology of the disease is poorly defined.

Neuroinflammation characterized by the activation of microglia, the neuroimmune cells of the CNS, is a key contributor of ICH-induced secondary brain injury and loss of neurological function (Wang and Dore, 2007; Carmichael et al., 2008; Wang, 2010). The introduction of blood components, including thrombin, Hb and Hb metabolites such as hemin into the brain creates the basis for neuroinflammatory responses after ICH (Wang and Dore, 2007; Carmichael et al., 2008; Robinson et al., 2009; Wang, 2010; Cai et al., 2011; Dang et al., 2011; Babu et al., 2012; Lin et al., 2012; Weng et al., 2015; Min et al., 2017). Notably, the proinflammatory activation of microglia after ICH correlates with blood-brain barrier damage, brain swelling/edema, hematoma expansion, neurological deterioration, and poor functional recovery (Platt et al., 1998; Hickenbottom et al., 1999; Leira et al., 2004; Zhao et al., 2007). Furthermore, inflammatory response after ICH also regulates the brain recruitment of blood-derived monocytes/macrophages that are known to regulate ICH-induced brain injury and thereby functional recovery (Tessier et al., 1997; Shiratori et al., 2010; Starossom et al., 2012).

Galectins are a family of evolutionary conserved carbohydrate-binding proteins (Barondes et al., 1994a,b; Kasai and Hirabayashi, 1996) involved in cell activation, differentiation, proliferation, migration and apoptosis (Perillo et al., 1995; Yang et al., 1996; Perillo et al., 1998; Moiseeva et al., 1999; Vespa et al., 1999; Yamaoka et al., 2000; Goldring et al., 2002). Among the various galectin family members, emerging evidences implicate key roles of Galectin-1 and Galectin-3 in neuroimmune responses in several neuropathological conditions (Jeon et al., 2010; Starossom et al., 2012; Parikh et al., 2015). However, there exists a critical knowledge gap in the understanding of their cellular expression and function after ICH. The objective of the current manuscript is to elucidate hemorrhagic-injury induced modulation and cellular expression of Galectin-1 and Galectin-3 expression in the brain in a preclinical model of ICH.

## Materials and Methods

### Induction of ICH

Intracerebral hemorrhage was induced in adult male CD-1 mouse (8–12 weeks; *n* = 43), as reported previously (Sukumari-Ramesh et al., 2012a,b, 2016; Bonsack et al., 2016; Sukumari-Ramesh and Alleyne, 2016; Ahmad et al., 2017; Chen-Roetling et al., 2017). Briefly, mouse was anesthetized (ketamine and xylazine) and a small incision was made to expose the skull. Using a high-speed drill, a burr hole (0.5 mm) was made on the skull approximately 2.2 mm lateral to bregma. Then the mouse was placed on to a stereotaxic head frame and a 26-G Hamilton Syringe was used to inject 0.04U of bacterial type IV collagenase (Sigma, St. Louis, MO, United States) in 0.5 μL Phosphate Buffer Saline (pH 7.4; PBS) into the left striatum (3.0 mm) under stereotaxic guidance. Upon removal of the needle, bone wax was used to seal the burr hole. Mice were kept at 37 ± 0.5°C using a small animal temperature controller throughout the procedure. The temporal pattern of hematoma after ICH is provided (Supplementary Figure S1).

### Western Blotting

Mice were anesthetized and transcardially perfused with PBS. Ipsilateral brain tissue (both hematomal and peri-hematomal brain regions) was collected in RIPA buffer containing protease and phosphatase inhibitors and subjected to sonication. The samples were then centrifuged at 14,000 rpm for 5 min at 4°C to collect the supernatant. Using a BCA protein assay kit (Pierce, Rockford, IL, United States) protein concentrations were estimated, and 30–50 micrograms of samples were run on a 4–20% sodium dodecyl sulfate–polyacrylamide gel and transferred onto a polyvinylidene difluoride (PVDF) membrane. Blots were incubated with the respective primary antibody, [Galectin-1 (1:1000,), R&D systems, Minneapolis, MN, United States), Galectin-3 (1:1000, Abcam, Cambridge, MA, United States), or β-actin (1:3000; Sigma, St Louis, MO, United States)] overnight at 4°C. This was followed by a 2-h incubation with a corresponding Alexa Fluor tagged secondary antibody. Blots were read using a Li-Cor Odyssey near-infrared imaging system and quantification was done using Quantity One software (Bio-Rad, Foster City, CA, United States).

### Immunohistochemistry

Mice were anesthetized and transcardially perfused with PBS. Brains were collected and placed in 4% paraformaldehyde overnight at 4°C, and then snap frozen. Brains were then cut into 25-mm coronal sections using a cryostat and mounted onto glass slides. Sections were incubated for 2 h in 10% normal donkey serum at room temperature. This was followed by an overnight incubation with the respective primary antibody at 4°C. After washing, the sections were incubated with the corresponding Alexa Fluor-tagged secondary antibody for 1 h at room temperature. Immunofluorescence was determined using a Zeiss LSM510 Meta confocal laser microscope and cellular co-localization was determined, as described earlier (Laird et al., 2010). We analyzed three non- consecutive sections per animal and a minimum of 3 random fields around the hematoma.

### Enzyme Linked Immunosorbent Assay

RAW 264.7, a murine macrophage cell line, were plated on a 24 well plate and allowed to incubate for 48 h in DMEM (Dulbecco’s Modified Eagle Medium) containing 5% Fetal Bovine Serum, 5% Bovine Growth Serum, and 1% Penicillin/Streptomycin. Cells were then incubated with mouse recombinant Galectin-1 (6.25 or 12.5 μg/ml; R&D Systems, Minneapolis, MN, United States) for 1 h and it was followed by an 18-h treatment with LPS (100 ng/ml) or hemin (30 μg/ml). The supernatant was collected and used for the detection of IL-6, by ELISA as per manufacturer’s instructions (Biolegend, San Diego, CA, United States). Briefly, a 96 well plate was coated overnight at 4°C with a specific capture antibody. Following a 1-h blocking, the cell culture supernatant was added to the wells and incubated for 2 h at room temperature. Any unbound materials were removed by washing and a detection antibody solution was added to the wells and allowed to incubate for 1 h at room temperature. After further washing, 100 μl of Avidin- Horse Radish Peroxidase (HRP) solution was added to each well for 30 min at room temperature. The substrate solution was added to the wells after washing for color development. A stop solution was used, and the plate was read at 450 nm using a microtiter plate reader (Bio-Tek, Epoch).

### Genetic Knockdown of Galectin-3

RAW 264.7 cells were transfected with either control siRNA (ON-TARGET plus Non-targeting Pool; GE Dharmacon) or Galectin-3 siRNA (ON-TARGET plus Mouse Lgals3 siRNA; GE Dharmacon) using HiPerFect Transfection Reagent (QIAGEN) according to manufacturer’s instructions. Galectin-3 knockdown was verified 48h post- transfection by western blotting as described earlier.

### Statistical Analysis

The data were analyzed using *t*-test or one-way analysis of variance followed by Student–Newman–Keuls *post hoc* test and was expressed as mean ± Standard Error (SE). A *p*-value of < 0.05 was considered to be significant.

## Results

### Temporal Expression Pattern of Galectin-1 and Galectin-3 After ICH

To evaluate whether hemorrhagic-injury results in modulation of Galectin-1 and Galectin-3 expression in the brain, ICH or Sham was induced in mice using the collagenase injection method. Given the emerging role of Galectin-1 and Galectin-3 in neuroimmune responses, the ipsilateral brain sections from sham or ICH mice were subjected to evaluation employing both western blotting and immunohistochemistry analysis at various time points ranging from day 1 through day 7 post surgery, a post-injury time period, which exhibited remarkable induction of both pro- as well as anti-inflammatory activation of microglia/macrophages as well as astrocytes after ICH (Sukumari-Ramesh et al., 2012b, 2016; Bonsack et al., 2016).

**FIGURE 1:**
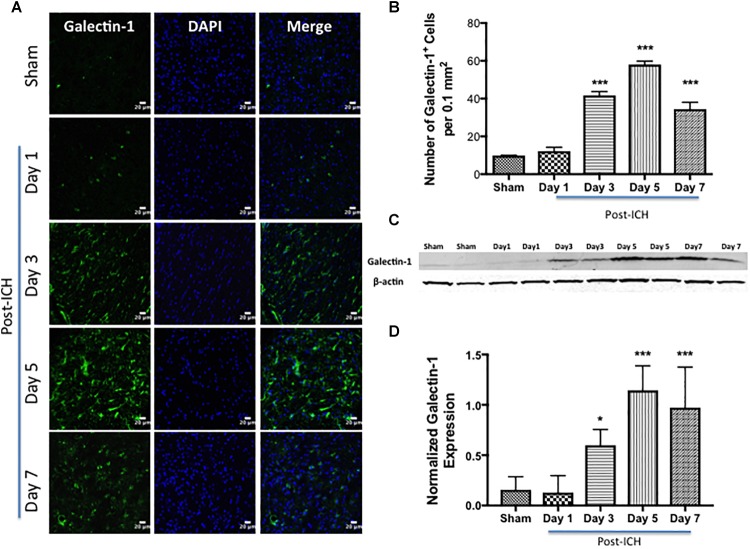
Increased expression of Galectin-1 after ICH. **(A)** Confocal images depicting the temporal expression pattern of Galectin-1 after ICH (scale bar = 20 μm; *n* = 3–5 per group). **(B)** The average number of Galectin-1-positive cells per 0.1 mm^2^ in the ipsilateral striatum. **(C)** Increased expression of Galectin-1 was confirmed by western blotting of brain tissue from the ipsilateral striatum. **(D)** Densitometry analysis (*n* = 3–5 per group) of the western blotting data. ^∗^*p* < 0.05, ^∗∗∗^*p* < 0.001 vs. sham.

**FIGURE 2:**
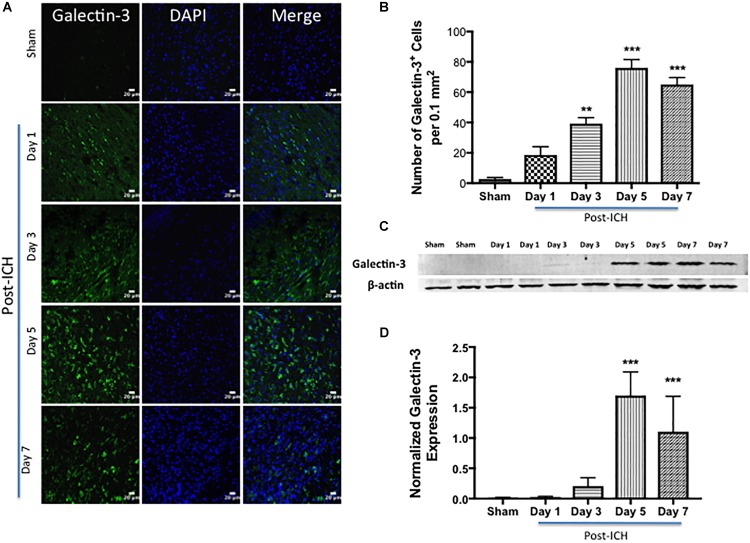
Augmented expression of Galectin-3 after ICH. **(A)** Temporal expression pattern of Galectin-3 in the perihematomal area after ICH. The representative confocal images show a remarkable increase in Galectin-3 expression after ICH in comparison to Sham (scale bar = 20 μm; *n* = 3–5 per group). **(B)** The average number of Galectin-3 positive cells per 0.1 mm^2^ in the ipsilateral striatum. **(C)** Temporal expression of Galectin-3 was further confirmed using western blotting. **(D)** Densitometry analysis (*n* = 3–5 per group) of the western blotting data. ^∗∗^*p* < 0.01, ^∗∗∗^*p* < 0.001 vs. sham.

Notably, brain sections from sham or contralateral brain areas from ICH exhibited very marginal or undetectable expression of Galectin-1 and Galectin-3 whereas augmented expression of Galectin-1 and Galectin-3 was observed at day 3, day 5, and day 7-post ICH (Figure 1, 2). Along these lines, the number of Galectin-1 immunopositive cells significantly increased by approximately 4, 6 and 4-fold on day 3, day 5, and day 7 post -ICH, respectively, in comparison to sham (Figure 1B). This observation was further validated using western blotting analysis, which revealed a remarkable and significant induction of Galectin-1 starting from day 3-post ICH in comparison to sham (Figure 1C,D). Further, the induction of Galectin-3 (Figure 2) mirrored the Galectin-1 expression after ICH and Galectin-3 immunopositive cells were approximately 15, 28 and 24- fold higher on day 3, day 5, and day 7 post-ICH (Figure 2B) in comparison to sham and the western blotting followed by densitometry analysis confirmed the injury-induced increased expression of Galectin-3 after ICH (Figure 2C,D).

### Cellular Localization of Galectin-1 and Galectin-3 After ICH

**FIGURE 3:**
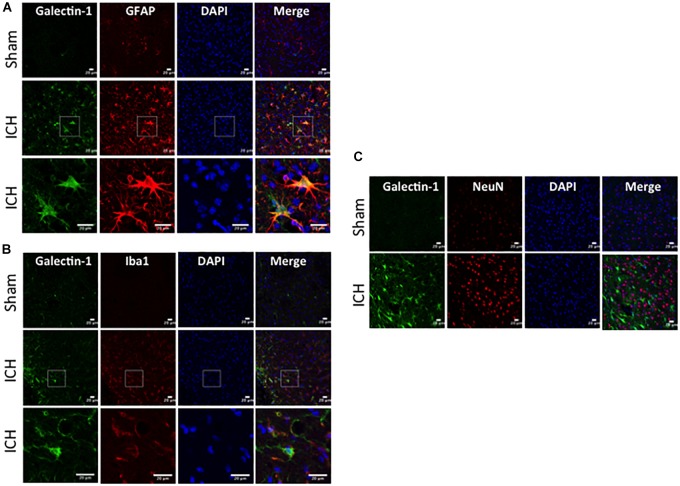
Galectin-1 expression is mostly observed in glial cells after ICH. Brain sections were double immunostained for **(A)** Galectin-1 and GFAP, **(B)** Galectin-1 and Iba1, (the lowest panel depicts the high magnification images) and **(C)** Galectin-1 and NeuN (Scale bar = 20 μm; *n* = 3 per group). Galectin-1 expression was observed mostly in GFAP-positive cells and in a subset of Iba1-positive cells. NeuN positive cells didn’t express Galectin-1 (*n* = 3 per group).

To determine the cellular localization of Galectin-1 and Galectin-3 after ICH, the brain sections were subjected to dual label immunostaining. Galectin-1 expression was mostly observed in GFAP-positive astrocytes (Figure 3A) and stereotactic cell counting revealed that 85% of Galectin-1 positive cells co-expressed GFAP after ICH. In addition, Galectin-1 expression was also observed in Iba1- positive microglia/macrophages after ICH (Figure 3B) but only 12% of Galectin-1 positive cells co-expressed Iba1.

**FIGURE 4:**
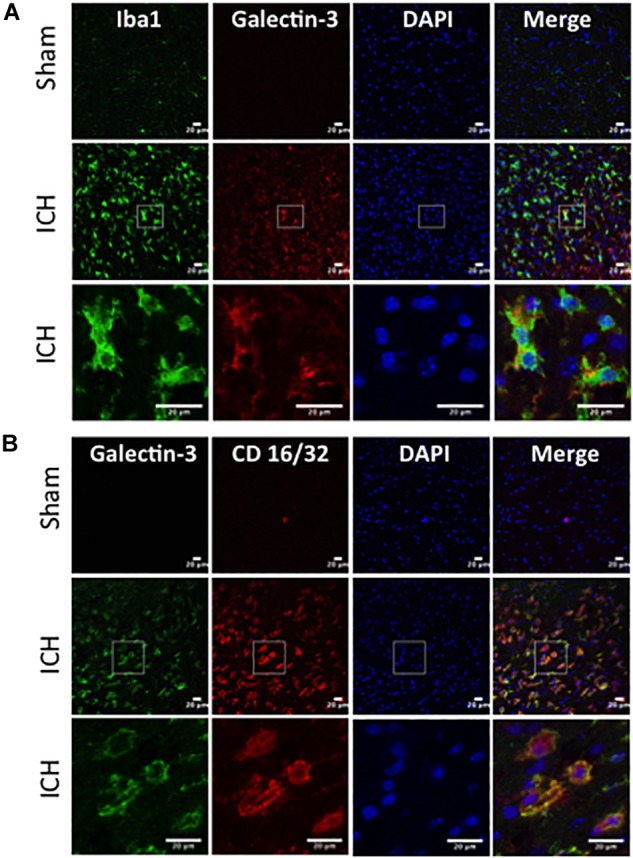
Galectin-3 expression in microglia/macrophages after ICH. Brain sections were immunostained for **(A)** Galectin-3 and Iba1, and **(B)** Galectin-3 and CD 16/32. Both Iba1 (a marker of microglia/macrophages) as well as CD16/32 (a marker of proinflammatory M1 microglia or macrophages)-positive cells, co expressed Galectin-3 (*n* = 3 per group).

**FIGURE 5:**
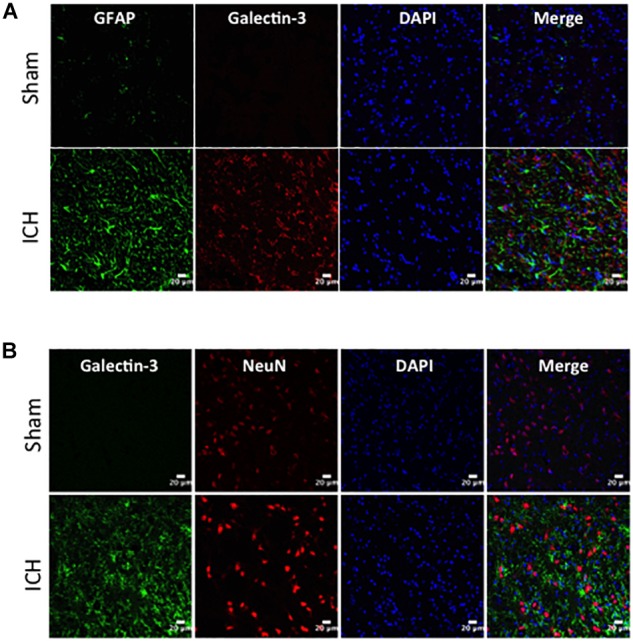
Cellular localization of Galectin-3 after ICH. Brain sections were double immunostained with **(A)** Galectin-3 and GFAP, and **(B)** Galectin-3 and NeuN. Galectin-3 expression was absent in either GFAP-positive or NeuN-positive cells. Scale bar = 20 μm; *n* = 3 per group.

In contrast, expression of Galectin-3 was mostly confined to Iba1-positive cells (Figure 4A) and Galectin-3 expression was absent in GFAP-positive cells (Figure 5A) indicating differential cellular expression of Galectin-1 and Galectin-3 after ICH. Further, the expression of Galectin-3 was observed in proinflammatory, M1 microglial or macrophage marker, CD16/32-positive cells (Figure 4B) implicating a novel role of Galectin-3 in neuroinflammatory responses after ICH. Notably, 88 and 92% of Galectin-3 positive cells coexpressed Iba1 and CD16/32-positive cells, respectively. Of note, Galectin-3 expressing microglia or macrophages exhibited phagocytic phenotype (Figure 4A,B) implicating its unexplored role in microglial or macrophage mediated phagocytosis after ICH. Further, NeuN-positive cells didn’t express either Galectin-1 or Galectin-3 (Figure 3C, 5B).

### Galectin-1 and Galectin-3 Mediated Regulation of Inflammatory Response

**FIGURE 6:**
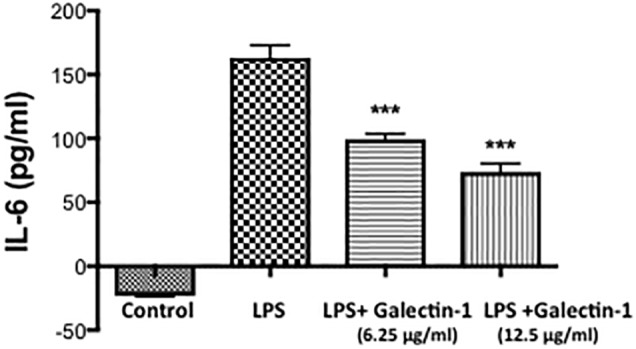
Recombinant Galectin-1 and inflammatory response. Prior to LPS (100 ng/ml) stimulation, Raw 264.7 cells were treated with recombinant Galectin-1, and the release of IL-6 was measured using ELISA, as detailed in methods. Recombinant Galectin-1 significantly reduced LPS-induced release of IL-6 from RAW 246.7 cells (*n* = 4 per group). ^∗∗∗^*p* < 0.001 vs. LPS.

To establish the possible functional role of Galectin-1 and Galectin-3 after ICH, we performed *in vitro* studies. Recombinant Galectin-1 (6.25 and 12.5 μg/ml) significantly attenuated LPS-induced release of a proinflammatory cytokine, Interleukin -6 (IL-6) from RAW 264.7 cells as estimated by ELISA in comparison to controls (Figure 6) implicating a negative regulatory role of Galectin-1 in inflammation.

**FIGURE 7:**
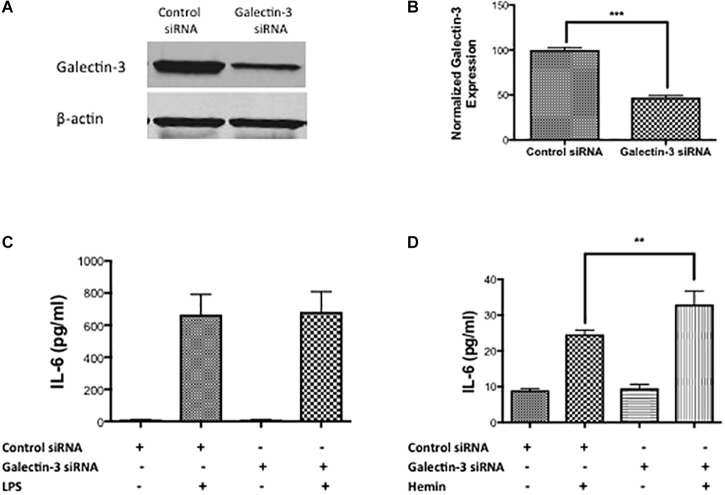
Galectin-3 and inflammatory response **(A)** RAW 246.7 cells were treated with either control siRNA or Galectin-3 siRNA as detailed in methods and the genetic knockdown of Galectin-3 was verified using **(A)** western blotting followed by **(B)** densitometry analysis. ^∗∗∗^*p* < 0.001 vs. control siRNA**. (C)** Galectin-3 knockdown didn’t modulate LPS-induced release of IL-6 **(D)** whereas significantly unregulated hemin–induced release of IL-6 from RAW 246.7 cells in comparison to control (*n* = 3 per group). ^∗∗^*p* < 0.01 vs. hemin.

To test the functional role of Galectin-3, we performed siRNA-mediated genetic knockdown of Galectin-3 in RAW 264.7 cells and examined the inflammatory response. Gene silencing with siRNA significantly reduced the Galectin-3 expression in RAW 264.7 cells by 52.95% as evidenced by western blotting (Figure 7A,B). Notably, siRNA medicated genetic knockdown of Galectin-3 did not modulate LPS-induced release of IL- 6 (Figure 7C) whereas the genetic knock down of Galectin-3 significantly augmented hemin (a hemoglobin metabolite that accumulates at high concentration in intracranial hematomas) –induced the release of IL-6 (Figure 7D) implicating an unexplored role of Galectin-3 in modulating inflammatory response after ICH.

## Discussion

Galectins are a family of endogenous carbohydrate-binding proteins that play critical roles in both physiological and pathological conditions by interacting with glycosylated receptors on the cell surface and modulating intracellular signaling pathways (Perillo et al., 1995, 1998; Yang et al., 1996; Moiseeva et al., 1999; Vespa et al., 1999; Yamaoka et al., 2000; Goldring et al., 2002; Laaf et al., 2019). Galectins exhibit significant sequence similarity in their carbohydrate-recognition domain (CRD) with an enhanced affinity toward β-galactosides and are originally defined by their ability to recognize the disaccharide *N*-acetyllactosamine (Barondes et al., 1994a,b; Kasai and Hirabayashi, 1996). However, recent studies demonstrate substantial differences in their carbohydrate binding properties (Hirabayashi et al., 2002; Leffler et al., 2002; Carlsson et al., 2007).

Galectin-1, the most ubiquitously expressed member of the galectin family (Stillman et al., 2006) has been implicated in the regulation of innate and adaptive immunity and is present in both intracellular and extracellular locations (Verschuere et al., 2014). The extracellular functions of Galectin-1 rely largely on the carbohydrate-binding properties while the intracellular functions involve mainly carbohydrate-independent interactions (Verschuere et al., 2014). Consistent with the role of Galectin-1 in immune response in the periphery, Galectin-1 is known to suppress macrophage activation (Barrionuevo et al., 2007), promotes selective apoptosis of T cells (Toscano et al., 2007), induces the secretion of anti-inflammatory cytokine, IL-10 (van der Leij et al., 2004; Cedeno-Laurent et al., 2012), and attenuates nitric oxide (NO) production by macrophages (Correa et al., 2003).

Galectin-1 is expressed widely in nervous tissues at embryonic stages but becomes restricted mainly to peripheral tissues upon maturation (Horie and Kadoya, 2002). Consistently, uninjured brain striatum exhibited marginal expression of Galectin-1. However, upon hemorrhagic brain injury very remarkable Galectin-1 expression was observed in GFAP-positive astrocytes. Along these lines, Galectin-1 is implicated in astrocyte differentiation and subsequent release of BDNF (Brain Derived Neurotrophic factor) after a brain injury implicating a role of Galectin-1 in neuroprotection (Sasaki et al., 2004; Qu et al., 2010). Further, Galectin-1 is one of the key regulators of adult neurogenesis through its carbohydrate-binding ability and promotes functional recovery after stroke (Ishibashi et al., 2007). Galectin-1 administration reduced apoptosis of neurons, decreased brain infarction volume and improved neurological function induced by brain ischemia (Qu et al., 2011). Also, native and recombinant galectin-1 protected mouse and rat cerebellar neurons from the neurotoxic effects of glutamate (Lekishvili et al., 2006). Of note, Galectin-1 deactivates inflammatory microglia and protects from inflammation-induced neurodegeneration (Starossom et al., 2012). Further, our studies demonstrated that recombinant Galectin-1 attenuates the release of a proinflammatory cytokine, IL-6 from LPS-stimulated murine macrophages, RAW 264.7 in comparison to controls implicating a negative regulatory role of Galectin-1 in inflammation.

Galectin-1 is one of the endogenous ligands of CD45 (Walzel et al., 1999), which regulates microglia/macrophage activation. In addition, Galectin-1 interaction with CD45 leads to the retention of this glycoprotein on the plasma membrane and augmenting its phosphatase activity. Recent studies demonstrated that CD45 negatively regulates proinflammatory M1 microglia activation but promotes anti-inflammatory, M2 phenotype through modulation of the mitogen-activated protein kinase p38 (p38MAPK), cAMP response element binding (CREB), and nuclear factor kappa-light-chain-enhancer of activated B cells (NF-kB) signaling pathways (Starossom et al., 2012). This effect involved binding of Galectin-1 to core 2 *O*-glycans on CD45 suggesting that the expression of glycan moieties on activated microglial/macrophages is required for Galectin-1 binding and function (Starossom et al., 2012). In addition, Galectin-1 suppressed methamphetamine-induced neuroinflammation in human brain microvascular endothelial cells (Parikh et al., 2015) and Galectin-1 is suggested to be involved in neurite outgrowth and synaptic connectivity. Altogether, the data suggest that Galectin-1 induction in reactive astrocytes after ICH could be an intercellular communication mechanism facilitating astrocyte-mediated regulation of neuroprotection after ICH warranting further investigation.

Galectin-3 is a 25–35 kDa chimeric type protein with functions tightly depend on the localization (Thomas and Pasquini, 2018). The expression of Galectin-3 has been found in the nucleus, and cytoplasm (Liu et al., 2002). Further, macrophages and activated microglia can release Galectin-3 in the extracellular space leading to extracellular matrix remodeling and altered inflammatory response, respectively (Li et al., 2008; Jeon et al., 2010). Instead of the classical endoplasmic reticulum/Golgi secretion pathway, Galectin-3 follows an alternative secretory pathway for secretion and export (Mehul and Hughes, 1997) and upon release, Galectin-3 interacts with several extracellular receptors. Though, Galectin-3 is closely linked to the inflammatory cascade of reactions; the precise functional role of Galectin-3 in neuroinflammation is largely controversial. However, it is reported that galectin-3 released by microglia acted as an endogenous TLR-4 (Toll Like Receptor-4) ligand (Burguillos et al., 2015). Further, the genetic deletion of Galectin-3 reduced neuronal loss and administration of Galectin-3 antibody exerted neuroprotective effects in a preclinical model of traumatic brain injury (Yip et al., 2017) together implicating a detrimental role of Galectin-3 after a brain injury. In contrast, targeted deletion of Galectin-3 exacerbated ischemic brain injury and neurodegeneration after cerebral ischemia (Lalancette-Hebert et al., 2012) suggesting a neuroprotective role of Gelectin-3 after brain damage. In addition, Galectin-3 contributes to angiogenesis and neurogenesis implicating its possible role in post-ischemic brain repair (Yan et al., 2009). Galectin-3 also promoted oligodendroglia differentiation, contributing to functional recovery following demyelinating disorders (Pasquini et al., 2011). These conflicting functional roles of Galectin-3 after neuropathology could be due to the differential subcellular expression of Galectin-3 or due to the difference in the pathophysiology of brain disorders warranting further investigation.

Consistent with other neuropathological conditions, we observed elevated expression of Galectin-3 after ICH and expression was predominantly observed in Iba1 positive cells, the inflammatory cells of the CNS. Iba1 positive cells after ICH could be either microglia or infiltrating macrophages, which play roles in innate immune response. Recent studies demonstrate that microglia and macrophages may have differential roles after brain pathology (Gao et al., 2017). Along these lines, studies with primary microglial culture document a proinflammatory role of Galectin-3 (Burguillos et al., 2015), whereas studies with macrophages demonstrate an anti-inflammatory role (MacKinnon et al., 2008) warranting further investigation. Moreover, genetic knockdown of Galectin-3 in RAW 246.7 cells augmented hemin- induced release of IL-6, a proinflammatory cytokine implicating a role of Galectin-3 in inflammatory responses after ICH. Besides, Galectin-3 expressing microglia or macrophages exhibited phagocytic phenotype implicating its unexplored role in microglial or macrophage mediated phagocytosis, which plays a key role in hematoma resolution and subsequent brain recovery after ICH. Consistently, recent reports suggest that macrophages that accumulate in the CNS during parasite infection abundantly express Galectin-3 (Quenum Zangbede et al., 2018) and activated microglia phagocytose cells via Galectin-3 (Nomura et al., 2017). In addition, elevated plasma Galectin-3 levels were strongly associated with inflammation, severity and poor outcomes in patients with acute ICH (Yan et al., 2016). Therefore, further studies are needed elucidating the functional roles of Galectin-3 after ICH.

## Conclusion

Galectin-1 and Galectin-3 exhibited very profound and increased expression from day 3 to day 7-post-injury, in the perihematomal brain region after ICH in comparison to Sham. Further, Galectin-1 expression was mostly observed in GFAP- positive astrocytes whereas Galectin-3 expression was observed mostly in Iba1 as well as CD16/32-positive cells, the inflammatory cells of the CNS. Moreover, genetic studies revealed a negative regulatory role of both Galectin-1 and Galectin-3 in the release of a proinflammatory cytokine, IL-6 depending on the stimulus. Altogether, the data suggest that Galectin-1 and Galectin-3 could be targeted in modulating glial responses and thereby brain injury after ICH, warranting further investigation.

## Ethics Statement

Animal studies were reviewed and approved by the Committee on Animal Use for Research and Education at Augusta University, in compliance with NIH and USDA guidelines.

## Author Contributions

FB carried out the immunohistochemical, and cell culture studies and western blotting and participated in the data analysis. SS-R conceived and designed the experiments, conducted the animal surgeries, genetic studies, and data analysis, and drafted the manuscript. Both authors read and approved the final manuscript.

## Conflict of Interest Statement

The authors declare that the research was conducted in the absence of any commercial or financial relationships that could be construed as a potential conflict of interest.

## Supplementary Material

FIGURE S1 The temporal pattern of hematoma after ICH.

## Abbreviations

CD16/32: Cluster of Differentiation 16/32
CD45: Cluster of Differentiation 45
CNS: central nervous system
ELISA: enzyme linked immunosorbent assay
GFAP: glial fibrillary acidic protein
Hb: hemoglobin
Iba1: ionized calcium binding adaptor molecule 1
ICH: Intracerebral Hemorrhage
LPS: lipopolysaccharide
NeuN: neuronal nuclei
PBS: phosphate buffer saline
RIPA: radioimmunoprecipitation.
