# Supplementary figures and images for: Differential Cellular Expression of Galectin-1 and Galectin-3 After Intracerebral Hemorrhage
Source: Front Cell Neurosci. 2019 May 14;13:157. doi: 10.3389/fncel.2019.00157 (PMC6530358; doi:10.3389/fncel.2019.00157)

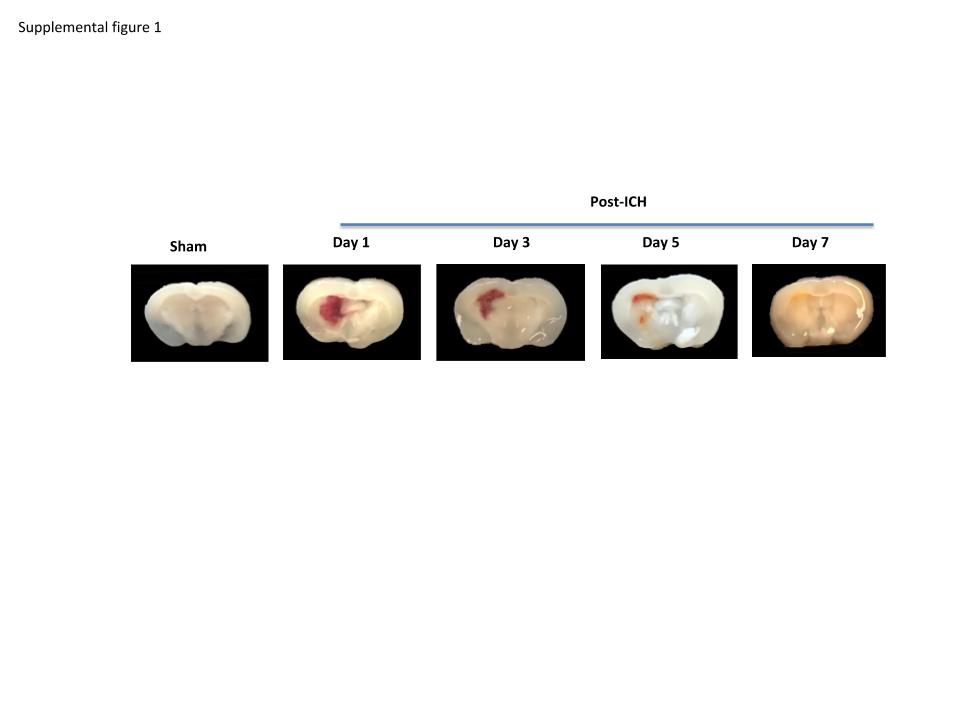

Supplement: FIGURE S1 — The temporal pattern of hematoma after ICH. [file Image_1.jpg]
